# Mitochondrial coupling efficiency and myofiber type related to blood pressure 22 h after high-intensity exercise in premenopausal women

**DOI:** 10.1007/s00421-025-05805-2

**Published:** 2025-05-10

**Authors:** Gary R. Hunter, Gordon Fisher, Stephen J. Carter, Douglas R. Moellering

**Affiliations:** 1 Department of Nutrition Sciences, School of Health Professions, University of Alabama at Birmingham, 1675 University Blvd, Susan Mott Webb Building Suite 439, Birmingham, AL 35233 USA; 2 Deparment of Human Studies, School of Education and Human Sciences, University of Alabama at Birmingham, Birmingham, AL 35294 USA; 3 Department of Kinesiology, School of Public Health, Indiana University Bloomington, Bloomington, IN 47405 USA; 4 Indiana University Melvin and Bren Simon Comprehensive Cancer Center, Indianapolis, IN 46202 USA

**Keywords:** Exercise training, Hypertension, Interval exercise, Respirometry, Skeletal muscle, Vascular health

## Abstract

Previously we have shown that systolic blood pressure (SBP) increases in African American (AA) women but decreases in European American (EA) women ≈22 h after a high-intensity exercise bout, suggesting delayed recovery in the AA women. We, therefore, sought to determine whether myofiber type, systemic vascular resistance (SVR), and mitochondrial coupling efficiency may contribute to elevated blood pressure in AA women following a bout of high-intensity exercise. Premenopausal EA (9) and AA (7) women were aerobically trained for 8–16 weeks and $\dot{V}{\text{O}}_{{{\text{2peak}}}}$ was evaluated. After 2 days without exercise, participants were evaluated for myofiber type, mitochondrial respiration using high-resolution respirometry, and SVR 22 h following 1 h of high-intensity interval cycle ergometry. AAs had higher SBP and DBP and type IIx myofiber % but lower type IIa myofiber %. SBP was significantly related to SVR (0.71), RCR (0.44), type IIa myofiber type (− 0.48), and type IIx myofiber type (0.53). DBP was significantly related to SVR (0.58) and the respiratory acceptor control ratio (state 3/state 4, termed RCR, 0.69). SBP remained significantly higher in AAs even after adjusting for type IIx myofiber type, RCR, SVR, or $\dot{V}{\text{O}}_{{{\text{2peak}}}}$ adjusted for FFM, and additionally, DBP remained significantly higher after adjusting for type IIx myofiber type, RCR, or $\dot{V}{\text{O}}_{{{\text{2peak}}}}$ adjusted for FFM. These results support the premise that mitochondrial RCR, type IIx myofiber type, and SVR may contribute to increased blood pressure ≈22 h following a bout of high-intensity exercise. Still, racial differences were not explained by any of these variables.

## Introduction

After 8–16 weeks of moderate-intensity aerobic training, corresponding to 40 min at 70% peak oxygen uptake ($\dot{V}{\text{O}}_{{{\text{2peak}}}}$), African American (AA) women exhibited an increased systolic blood pressure (SBP) ≈22 h after an acute, unaccustomed bout of high-intensity interval exercise (Carter et al. 2016). This observation contrasted with the decreased SBP measured in European American (EA) women. Among all participants, the changes in SBP were related to changes in small artery elasticity, an index of endothelial function (Grey et al. 2003; McVeigh et al. 2001). A possible explanation for this divergent response is that the AA women may not have been fully recovered after the HII exercise (Hunter et al. 2018). Delayed recovery may work in concert with systemic inflammation, which, in turn, may elicit undesirable transient neuro-hormonal changes affecting vascular resistance and central fatigue (Carfagno and Hendrix 2014).

Type II myofiber may be related to increased blood pressure (Hall et al. 2021). Perhaps, at least partially due to lower capillary density and thus increased arterial resistance in type II myofiber. In addition, it is possible that type II myofiber, especially fatigable type IIx myofibers, may be slower to recover from high-intensity exercise. Supporting this contention, type II myofibers (Quindry et al. 2011) as well as the percentage of fast-twitch myofibers, albeit in animal models, are associated with more oxidative stress following exercise training (Chang et al. 2014).

Although multiple factors contribute to the development of hypertension, chronic inflammation plays an important role (Usui 2023). Exercise training is known to decrease systemic inflammation but a bout of unaccustomed high-intensity exercise has been linked to increased systemic inflammation and formation of reactive oxygen species (ROS) (Kolodziej and O’Halloran 2021). One mechanism to offset the formation of ROS after an acute bout of high-intensity exercise is activation of uncoupling proteins. Uncoupling proteins are a class of mitochondrial carrier proteins that dissipate the proton gradient generated by the electron transport system. This uncouples oxidative phosphorylation, which increases metabolic rate (Jevtovic et al. 2022). Some studies have found differences in uncoupling protein 3 (UCP3) activity between black and white people linked to resting energy expenditure (Kimm et al. 2002) and body composition (Lanouette et al. 2002). Paradoxically, sedentary behavior is also associated with increased oxidative stress and systemic inflammation (Kolodziej and O’Halloran 2021). According to the *“uncoupling to survive”* theory, a small increase in mitochondrial uncoupled respiration is thought to reduce ROS formation and have a mediating effect on metabolic health including increased blood pressure (Klaus and Ost 2020).

Low resting energy expenditure may in part be mediated by a higher mitochondrial coupling efficiency. More of the energy derived from oxygen consumption is used to produce ATP with less ‘wasted’ energy that would be dissipated as heat through increased mitochondrial proton leak. This person would have a relatively low state 4 respiration (mitochondrial proton leak) or low state 3 respiration/state 4 respiration ratio (termed the respiratory acceptor control ratio or RCR) (Chance and Williams 1955b, a, 1956). It is well-established that AAs have lower resting energy expenditures than EAs (Weinsier et al. 2000). Although several potential causes for these differences have been identified (Hunter et al. 2000; Wang et al. 2010), no one has definitively identified the total cause for these differences. One possibility could be differences in mitochondrial uncoupling activity. If AAs tend to exhibit higher mitochondrial coupling efficiency, it is possible this would coincide with less uncoupling activity and lower resting energy expenditure—potentially contributing to increased systemic inflammation, oxidative stress, and blood pressure.

Therefore, the purpose of this paper is to determine if blood pressure following high-intensity exercise is higher in AA women. In addition, we hypothesize that racial differences in blood pressure are mediated by myofiber type, systemic vascular (SVR) resistance, and uncoupled mitochondrial respiration. Finally, it is also hypothesized that the relationship between myofiber type and blood pressure is mediated by differences in SVR and increased RCR.

## Methods

### Participants

Nine EA and seven AA premenopausal women 20–40 y participated in this study. Participants reported normal menstrual cycles and were not taking oral contraceptives or medications that influence metabolism. In addition, participants were: (1) normotensive; (2) non-smokers; (3) sedentary, as defined by exercise training < 1 × per week; and (4) normoglycemic as evaluated by postprandial glucose response to a 75-g oral glucose tolerance test. Written informed consent was supplied to all participants. Approval was obtained from the Institutional Review Board at the University of Alabama at Birmingham and conformed to the guidelines set forth by the Declaration of Helsinki.

### Design

**Fig. 1:**
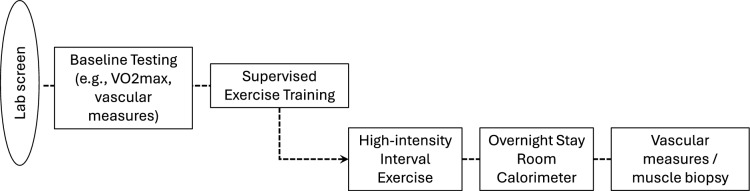
Schematic showing the overall study design

This is a secondary evaluation of a study designed to evaluate post-exercise training changes in insulin sensitivity (Fisher et al. 2019). Participants were evaluated four times during the follicular phase of four different menstrual cycles after the initial screening and fitness assessments. Prior to testing, participants stayed in a room calorimeter for 23 h. Only the post-high-intensity trial is reported in this paper. After ~ 8–16 weeks of exercise training, post-training evaluations took place, ≈22 h after 1 h of interval stationary cycle ergometry at 84% $\dot{V}{\text{O}}_{{{\text{2peak}}}}$. Work intervals for the HII condition were 144 s followed by 103 s stationary rest intervals (work: rest intervals). Baseline and post-training assessments were performed 1 month apart. Figure 1 depicts a flow chart outlining the study design.

### Food intake

Food was provided 24 h prior to the room calorimeter visit and during the day spent in the room calorimeter. Diets were prepared by the Clinical Research Unit kitchen staff and consisted of ≈60% energy as carbohydrates, ≈25% as fat, and ≈15% as protein.

### Energy balance clamp

One goal was to try to achieve energy balance (energy intake matching energy expenditure) during the stay in the room calorimeter. Initial food requirements for the day preceding the room calorimetry visit were based on data accrued from 330 experiments using doubly-labeled water to estimate free-living energy expenditure of sedentary premenopausal women collected in our lab.

1 $${75}0 {\text{kcal}}\, + \,[\left( {{31}.{\text{47 FFM}}} \right)\, - \,\left( {0.{31}\, \times \,{\text{fat mass}}} \right)\, - \,\left( {{155}\, \times \,{\text{race }}\left( {\text{race coded 1 for African American and 0 for European American}} \right)} \right]$$

An equation for estimating the room calorimeter energy intake was developed from 200 room calorimeter visits of premenopausal women.

2 $${465} {\text{kcal}}\, + \,[\left( {{27}.{\text{8 FFM}}} \right)\, - \,\left( {{2}.{4}\, \times \,{\text{fat mass}}} \right)\, - \,\left( {{188}\, \times \,{\text{race }}\left( {{\text{race coded 1 for African American and }}0{\text{ for European American}}} \right)} \right]$$

Based on the anticipated energy expenditure of the exercise during the HII visit, the estimated energy cost of the exercise was added to Eq. 2 result:

3 $$({\text{Eq}}. {2}\;{\text{estimated energy expenditure}}\, + \,{\text{energy cost of HII exercise}})$$

However, we recognized that the estimates may result in overfeeding or underfeeding individual participants. Therefore, we developed a correction equation for the room calorimeter visit that was based on energy expenditure during the room calorimeter stay up to 1730. This equation was

4 $${.9}\left( {{39}0 \;{\text{kcal}}\, + \,{\text{average energy expenditure in kcal}}/{\text{min}}\;{\text{between }}0{8}00\;{\text{and 173}}0} \right)\, \times \,({925} {\text{kcal}}){-}{\text{Eq}}. {\text{3 estimate of energy expenditure}}]$$

We then adjusted the food intake of the evening meal to match the results of Eq. 4. The energy balance clamp was deemed affective given the absence of a significant difference between energy burned and energy consumed during the room calorimeter stay (given 2231 ± 256 and burned 2260 ± 268; *p* = 0.25).

### ***Peak ***$\dot{V}{\text{O}}_{{{\text{2}}}}$

Two to four days prior to each room calorimeter visit, $\dot{V}{\text{O}}_{{{\text{2peak}}}}$ was measured. After warm-up, participants completed a graded cycle ergometer test to measure peak oxygen uptake (V̇O_2peak_) as determined by the highest level reached in the final stage of exercise. Power output began at 25 W and increased by 25 W every 2 min until participants reached volitional exhaustion. Sixty RPM cycle cadence was maintained throughout the test. Oxygen uptake, ventilation, and respiratory exchange ratio were determined by indirect calorimetry using a MAX-II metabolic cart (Physio-Dyne Instrument Company, Quogue, NY). Heart rate was continuously monitored by Polar^®^ Vantage XL heart rate monitors (Polar Beat, Port Washington, NY). Criteria for achieving a true maximum were heart rate within 10 beats of estimated maximum, respiratory exchange ratio of at least 1.10, and plateauing. All participants reached at least one criterion and all but three participants reached at least two criteria at each of the four test time points.

### Supervised exercise training

After the baseline testing, all participants aerobically trained three times/week on a cycle ergometer for 12 weeks. Initial training was for 20 min at 65% of maximum heart rate. Intensity and volume were gradually increased until participants were training continuously for 40 min at 80% of maximum heart rate during week four. Heart rate was monitored throughout each session, Polar Vantage XL heart rate monitor (Polar Beat, Port Washington, NY). An exercise physiologist supervised all sessions.

### Room calorimeter

Participants spent 23 h in a whole-room respiration calorimeter (3.38 m long × 2.11 m wide × 2.58 m high) for measurement of total energy expenditure and REE. The design characteristics and calibration of the calorimeter were described previously (Treuth et al. 1996). Oxygen consumption and carbon dioxide production were continuously measured with the use of a magnetopneumatic differential oxygen analyzer (Magnos206; ABB, Frankfurt, Germany) and a nondispersive infrared industrial photometer differential carbon dioxide analyzer (Uras26, ABB, Frankfurt, Germany). The calorimeter was calibrated before each participant entered the chamber. Prior to each test, calibration was carried out on the oxygen and carbon dioxide analyzers using standard gases. The full scale was set for 0–1% for the carbon dioxide analyzer and 0–2% for the oxygen analyzer. Each participant entered the calorimeter at 0800 h. Metabolic data were collected throughout the 23 h stay. Each participant was awakened in the calorimeter at 0630 the next morning.

### Exercise sessions in room calorimeter

American College of Sports Medicine metabolic equations were used to determine workload on the Collins electronically braked cycle ergometer. Work intervals for the HII exercise were at 84% $\dot{V}{\text{O}}_{{{\text{2peak}}}}$ for 144 s with rest intervals of 103 s. Workload was remotely controlled outside the room calorimeter via a cable interface with the Collins electronically braked cycle ergometer (Warren E. Collins, Braintree, MA).

### Body composition

Fat mass and lean mass were determined by dual energy X-ray absorptiometry (DXA; iDXA, GE-Lunar, Madison, WI). Participants were evaluated fasted 3 to 4 days prior to the room calorimeter visit in compliance with manufacture recommended testing procedures. Scans were analyzed by the same investigator with ADULT software, LUNARDPX-L version 1.33 (GE Medical Systems Lunar).

### Tissue biopsy and preparation of permeabilized myofibers

Twenty-two hours following the exercise challenge, a subset of 17 women had muscle biopsies sampled (70–140 mg) from the vastus lateralis. Muscle biopsy samples were obtained from the lateral side of the vastus lateralis under local subcutaneous anesthesia (1% lidocaine) by percutaneous needle biopsy using a 5-mm Bergstrom needle under suction, as previously described (Fisher et al. 2019). Each of the four biopsies were performed on the contralateral leg based on the prior biopsy and subsequent biopsies on the same leg were performed a minimum of 1-inch distance from the prior biopsy with a minimum of 8 weeks in between. A portion of the biopsy sample was immediately placed and transported in an ice-cold relaxing and preservation solution BIOPS, containing (in mM) 2.77 Ca-EGTA buffer, 0.0001 free calcium, 50 K-MES, 7.23 K2EGTA, 20 imidazole, 0.5 DTT, 20 taurine, 5.7 ATP, 14.3 PCr, and 6.56 MgCl2-6 H2O (pH 7.1, 290 mOsm) (27; 33) and was used to prepare permeabilized myofiber bundles (PmFB). Briefly, small pieces of skeletal muscle (~ 20– 25 mg) were placed immediately in fresh ice-cold BIOPS, trimmed of fat and connective tissue on ice, and separated into four small muscle bundles (~ 2–6 mg. wet weight). The PmFBs were mechanically separated by gentle blunt dissection with a pair of needle-tipped, anti-magnetic forceps under magnification (Zeiss, Stemi S2000-C Stereo Microscope, Diagnostic Instruments). They were then treated with 30 µg/ml saponin, gently rocked (Rocker II, model 260,350, Boekel Scientific) at 4 °C for 30 min in BIOPS.]. PmFBs were then rinsed twice by gentle rocking to wash out saponin and ATP at 4 °C for at least 15 min and < 30 min, in MiR05 containing (in mM) 105 K-MES, 30 KCl, 1 EGTA, 10 K2HPO4, and 5 MgCl2-6 H2O, with 0.5 mg/ml BSA (pH 7.1, 290 mOsm). The PmFBs were then transferred to a fresh MiR05/creatine solution (500 µl) and blebbistatin (BLEB, 25 µM).

### High-resolution mitochondrial respirometry in permeabilized fibers

Mitochondrial respiration assays were performed using high-resolution respirometry by measuring oxygen consumption in 2 mL of MiR05/creatine/blebbistatin buffer, in a two channel respirometer (Oroboros Oxygraph-2 k with DatLab software; Oroboros Instruments Corp., Innsbruck, Austria) with constant stirring at 750 rpm (28) and following a modified substrate-uncoupler-inhibitor titration (SUIT) protocol to evaluate respiratory control in a sequence of coupling and inhibitory states induced by multiple titrations in each assay (34). Seventy percent ethanol was run in both chambers for a minimum of 30 min, rinsed 3 × with Milli-Q ultrapure ddH2O, and the chambers were calibrated after a stable air-saturated signal was obtained before every experiment. Reactions were conducted with PmFB at 37 °C with hyper-oxygenation to maintain oxygen concentrations above air saturation (~ 500–200 µM) (21) and prevent oxygen diffusion restrictions which have been shown to limit oxygen supply to the core of the fiber bundle (17). All experiments were completed in the Oxygraph chamber with [O2] concentrations above 150 µM. Respiration rates were measured using 4 mM malate, 9 mM pyruvate, and 2.5 mM succinate to drive convergent electron input to complexes I and II of the Electron Transport System (ETS). Polarographic oxygen measurements are expressed as pmol·s^−1^·mg^−1^ wet wt. Polarographic oxygen measurements are expressed as picomole per minute per milligrams wet weight. Determination of state 2, 3, and 4 respiration rates was made in the presence of substrate alone (state 2; LEAK state; low ATP), after the addition of ADP (2 mM; State 3; OxPhos State), and after inhibition of ATP synthase (Complex V) with oligomycin (State 4; LEAK State; high ATP). For quality control and to ensure outer mitochondrial membrane integrity, cytochrome c (10 µM) was added to the assay after activation by ADP and only preparations with < 10% increase after addition were included. Respiratory acceptor control ratios (RCRs) were determined as the ratio of State 3/State 4 respiration rates.

### Myofiber type

All visible connective and adipose tissues were removed from the biopsy samples with the aid of a dissecting microscope. Portions used for immunohistochemistry were mounted cross-sectionally on cork in optimum cutting temperature mounting medium mixed with tragacanth gum, frozen in liquid nitrogen-cooled isopentane, and stored at − 80 °C. The relative distribution of myofiber types I, IIa, and IIx were determined by myosin heavy chain immunohistochemistry using our well-established protocol (Kim et al. 2005).

### Rested blood pressure, systemic vascular resistance and cardiac output

Blood pressure, SVR, cardiac output (CO), and stroke volume (SV) were measured using non-invasive, local pulse contour analysis (HDI/Pulse Wave TM CR-2000, Hypertension Diagnostics Inc., Eagan, MN). Pulse contour analyses from the radial artery are based on a modified Windkessel model, allowing the evaluation of microcirculatory vessels (Cohn et al. 1995). In short, participants were seated and quiet during the testing. A blood pressure cuff was placed on the non-dominant arm. A solid-state pressure transducer was fastened over the radial artery of the dominant arm. The sensor was adjusted to achieve the highest relative signal strength. Assessments were made in triplicate and averaged. SVR was determined by analyzing a 30-s analog tracing of the radial waveform digitized at 200 samples per second. As previously described (Cohn et al. 1995), a beat determination was constructed using a beat-marking algorithm which determined the beginning systole, peak systole, onset of diastole, and end diastole during the 30-s period. The beat was incorporated into a parameter estimating algorithm (Hypertension Diagnostics Inc., Eagan, MN) and a modified Windkessel model was used to estimate SVR.

### Statistical analyses

Univariate analyses of variance (ANOVA) was used to compare between-group differences. Partial eta^2^ effect size was calculated to estimate the proportion of variance accounted for by the main effects. Separate univariate ANOVA was also run comparing SBP and DBP between AA and EA women after adjusting for potential confounders: type IIx %, RCR, SVR, $\dot{V}{\text{O}}_{{{\text{2peak}}}}$ adjusted for FFM. Pearson-product correlations were calculated between all pertinent variables. Separate multiple regression estimates for SBP and DBP were determined using either type IIx fiber type and RCR, type IIa fiber type and RCR, type IIx fiber type and SVR, or type IIa type and SVR. IBM SPSS statistics 29.0 was used to analyze.

## Results

Table 1 contains comparisons between EAs and AAs for variables of interest. A significant difference was observed with AA having higher SBP, DBP and SV but lower $\dot{V}{\text{O}}_{{{\text{2peak}}}}$ (both adjusted for body mass and adjusted for FFM) than EA. AA women had non-statistically significant greater body weight and BMIs but there were no observed differences for % fat. AA also had statistically significant lower type IIa myofiber % and higher type IIx myofiber % compared to EA but there was no significant difference in type I myofiber between groups. No other significant differences were found between AA and EA women.

**Table 1:** Between-group comparisons of European Americans and African Americans 22 h after a high-intensity interval exercise

	European Americans (*n* = 9)	African Americans (*n* = 7)	*p* value	Partial eta^2^
Age (y)	31.8 ± 7.6	33.8 ± 4.2	0.38	0.05
$\dot{V}{\text{O}}_{{{\text{2peak}}}}$ (mL/kg /min)	27.9 ± 4.5	22.3 ± 3.7	< 0.02	0.33
$\dot{V}{\text{O}}_{{{\text{2peak}}}}$ FFM (mL/kg FFM/min)	46.5 ± 7.6	36.1 ± 5.1	< 0.02	0.39
SBP (mm Hg)	115 ± 11	136 ± 13	< 0.01	0.49
DBP (mm Hg)	72 ± 11	81 ± 10	< 0.05	0.19
Body fat (%)	39 ± 6	38 ± 4	0.65	0.02
Mass (kg)	74.1 ± 12.9	77.4 ± 12.7	0.62	0.02
Height (cm)	165.6 ± 3.3	164.3 ± 3.2	0.44	0.04
BMI (kg/m^2^)	26.8 ± 4.9	29.0 ± 4.4	0.37	0.05
SVR (dyne/s/cm^−5^)	1365 ± 196	1513 ± 267	0.24	0.11
RCR	2.3 ± 0.6	2.4 ± 0.8	0.81	0.01
State 3 respiration	23.5 ± 12.6	18.5 ± 4.1	0.37	0.07
State 4 respiration	12.1 ± 4.9	8.2 ± 2.2	0.10	0.21
Type I myofiber %	32.1 ± 6.9	33.9 ± 4.2	0.80	0.01
Type IIa myofiber %	48.9 ± 10.2	39.9 ± 6.2	< 0.03	0.23
Type IIx myofiber %	19.0 ± 10.9	27.1 ± 5.7	< 0.05	0.19

Table 2 contains correlations of variables of interest. SBP and DBP were highly related to each other (*r* > 0.85). SBP was negatively related to type IIa myofiber type and positively related to type IIx myofiber type, while DBP was related to neither fiber type. SBP and DBP were positively related to both SVR and RCR. CO was negatively related to SVR and positively related to SV. SVR was only related to Type IIx myofiber type.

**Table 2:** Correlation coefficients among variables 22 h after high-intensity interval exercise

	DBP	SVR	$\dot{V}{\text{O}}_{{{\text{2peak}}}}$ Adj FFM	RCR	Type I	Type IIa	Type IIx
SBP	0.85** *N* = 17	0.71** *N* = 15	− 0.37 *N* = 15	0.44* *N* = 13	− 0.10 *N* = 16	− 0.48* *N* = 16	0.53* *N* = 16
DBP		0.58** *N* = 15	− 0.30 *N* = 15	0.69** *N* = 13	− 0.33 *N* = 16	− 0.08 *N* = 16	0.27 *N* = 16
SVR			− 0.31 *N* = 14	0.51* *N* = 13	0.13 *N* = 15	− 0.19 *N* = 15	0.11 *N* = 15
$\dot{V}{\text{O}}_{{{\text{2peak}}}}$Adj FFM				− 0.25 *N* = 12	0.11 *N* = 15	0.25 *N* = 15	− 0.28 *N* = 17
RCR					− 0.49 *N* = 13	0.26 *N* = 13	0.03 *N* = 13
Type I						− 0.27 *N* = 17	− 0.32 *N* = 17
Type IIa							− 0.83** *N* = 17

*SBP* systolic blood pressure, *DBP* diastolic blood pressure, *MAP* mean arterial blood pressure, *SVR* systemic vascular resistance, $\dot{V}{\text{O}}_{{{\text{2peak}}}}$ adj FFM, *RCR* state 3 mitochondrial respiration/state 4 mitochondrial respiration, *type I* type I myofiber %, *type IIa* type IIa myofiber %, *type IIx* type IIx myofiber %

Table 3 contains four separate SBP racial comparisons after adjusting for potential confounders type IIx %, RCR, SVR, $\dot{V}{\text{O}}_{{{\text{2peak}}}}$ adjusted for FFM, CO, and SV. All estimates of SBP were very similar to the unadjusted SBP comparisons with all still showing that AA women had significantly higher SBP than EA women. Table 4 contains four separate DBP racial comparisons after adjusting for potential confounders type IIx %, RCR, SVR, $\dot{V}{\text{O}}_{{{\text{2peak}}}}$ adjusted for FFM. All estimates of DBP were very similar to the unadjusted DBPs with all still showing that AA women had significantly higher DBP than EA women except for when the DBP contrast was adjusted for CO or SV.

**Table 3:** Multiple linear regression for systolic blood pressure

	EA	AA	*p* value	Partial eta^2^
Adjusted for type IIx %	115 ± 9	136 ± 3	< 0.02	0.38
Adjusted for RCR	115 ± 11	138 ± 12	< 0.01	0.59
Adjusted for SVR	115 ± 9	135 ± 13	< 0.02	0.42
Adjusted for $\dot{V}{\text{O}}_{{{\text{2peak}}}}$ Adj FFM	114 ± 10	136 ± 12	< 0.01	0.46

**Table 4:** Multiple linear regression for diastolic blood pressure

	EA	AA	*p* value	Partial eta^2^
Adjusted for type IIx %	72 ± 11	82 ± 10	< 0.18	0.13
Adjusted for RCR	74 ± 12	82 ± 10	< 0.01	0.53
Adjusted for SVR	72 ± 11	80 ± 10	0.42	0.06
Adjusted for $\dot{V}{\text{O}}_{{{\text{2peak}}}}$ Adj FFM	69 ± 6	81 ± 9	< 0.03	0.35

**Fig. 2:**
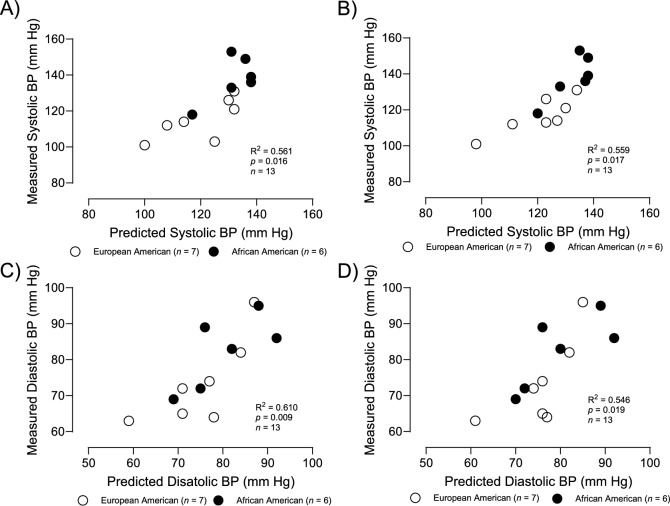
Scatterplots illustrating the relationship between: **A** measured SBP and estimated SBP using type IIx myofiber % (partial *r* = 0.70, *p* < 0.01) and carbohydrate RCR (partial 0.47 and *p* = 0.06); **B** Scatterplot illustrating the relationship between measured SBP and estimated SBP using type IIa myofiber% (partial *r* = − 0.70, *p* < 0.01) and carbohydrate RCR (partial 0.62, *p* < 0.03); **C** scatterplot illustrating the relationship between measured DBP and estimated DBP using type IIx myofiber% (partial *r* = 0.53, *p* < 0.04) and carbohydrate RCR (partial *r* = 0.73, *p* < 0.01); **D** scatterplot illustrating the relationship between measured DBP and estimated DBP using type IIa myofiber% (partial *r* = − 0.43, *p* < 0.11) and carbohydrate RCR (partial *r* = 0.74, *p* < 0.01)

Scatterplot in Fig. 2A illustrates the relationship between measured SBP and estimated SBP using type IIx myofiber % and carbohydrate RCR (*R*^2^ = 0.56 and *p* < 0.02). Scatterplot in Fig. 2B illustrates the relationship between measured SBP and estimated SBP using type IIa myofiber% and carbohydrate RCR (*R*^2^ = 0.56 and *p* < 0.02). Scatterplot in Fig. 2C illustrates the relationship between measured DBP and estimated DBP using type IIx myofiber% and carbohydrate RCR (*R*^2^ = 0.61 and *p* < 0.01). Scatterplot in Fig. 2D illustrates the relationship between measured DBP and estimated DBP using type IIa myofiber% and carbohydrate RCR (*R*^2^ = 0.55 and *p* < 0.02).

**Fig. 3:**
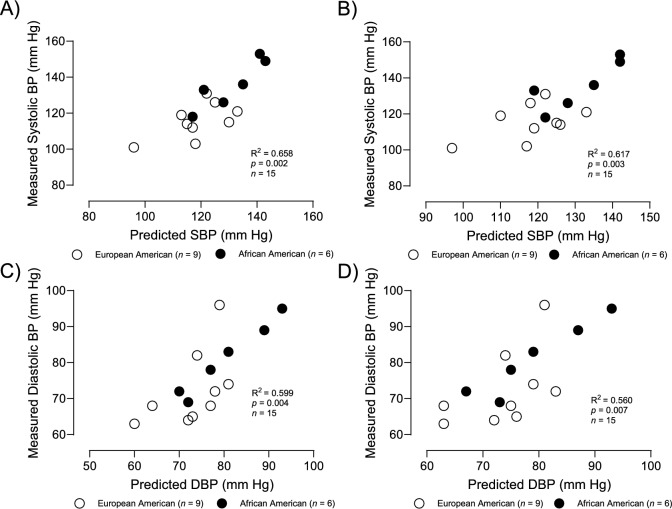
Scatterplots illustrating the relationship between: **A** measured SBP and estimated SBP using type IIx myofiber % (partial *r* = 0.64 and *p* < 0.02) and SVR (partial *r* = 0.71 and *p* = 0.01); **B** scatterplot illustrating the relationship between measured SBP and estimated SBP using type IIa myofiber % (partial *r* = − 0.58, *p* < 0.03) and SVR (partial *r* = 0.67, *p* < 0.01); **C** scatterplot illustrating the relationship between measured DBP and estimated DBP using type IIx myofiber % (partial *r* = 0.28, *p* = 0.30) and SVR (partial *r* = 0.75, *p* < 0.01): **D** scatterplot illustrating the relationship between measured DBP and estimated DBP using type IIa myofiber % (partial *r* = − 0.2, *p* = 0.94) and SVR (partial *r* = 0.74, *p* < 0.01)

Scatterplot in Fig. 3A illustrates the relationship between measured SBP and estimated SBP using type IIx myofiber % and SVR (*R*^2^ = 0.65 and *p* < 0.01). Scatterplot in Fig. 3B illustrates the relationship between measured SBP and estimated SBP using type IIa myofiber% and SVR (*R*^2^ = 0.62 and *p* < 0.01). Scatterplot in Fig. 3C illustrates the relationship between measured DBP and estimated DBP using type IIx myofiber% and SVR (*R*^2^ = 0.60 and *p* < 0.01). Scatterplot in Fig. 3D illustrates the relationship between measured DBP and estimated DBP using type IIa myofiber% and SVR (*R*^2^ = 0.56 and *p* < 0.01).

**Fig. 4:**
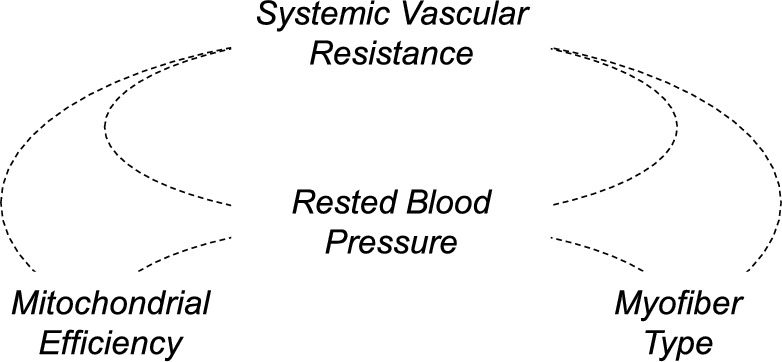
Theoretical model illustrating the interlinking relationships between systemic vascular resistance, rested blood pressure, mitochondrial efficiency, and myofiber type

Figure 4 illustrates a theoretical model showing that mitochondrial efficiency, systemic vascular resistance, and myofiber type are interrelated with resting blood pressure, potentially participating in the regulation of resting blood pressure.

## Discussion

As hypothesized, blood pressure ≈22 h following high-intensity biking was higher and aerobic capacity was lower in AA women than in EA women, even after 12 weeks of aerobic training. In addition, AA women had a higher % type IIx myofiber and lower % type IIa myofiber. Blood pressure was positively associated with type IIx and negatively associated with type IIa myofiber %, as well as RCR and arterial resistance. However, none of these factors explained the racial differences in BP.

It is interesting that RCR is related to blood pressure even after adjusting for race (SBP partial *r* = 0.57, and DBP partial *r* = 0.55). One possible explanation for these relationships may be found with Brand’s *“uncouple to survive”* theory. He proposes that one explanation for mitochondrial uncoupled respiration (that averages over 25% of mitochondrial respiration) is that this proton leak may protect against excessive production of superoxide and other reactive oxygen species, which in turn could decrease oxidative damage (Brand 2000). Since oxidative stress might be expected to increase vascular resistance, the positive relationship between vascular and mitochondrial uncoupling (carbohydrate RCR) is supportive of this theory. The increased vascular resistance would also be expected to increase blood pressure; thus, the positive association between vascular resistance and both SBP and DBP further supports Brand’s *“uncouple to survive theory”*. Taken together, these data support the possibility that type IIx myofibers may contribute to a delayed recovery from a high-intensity exercise bout contributing to increased oxidative stress, increased vascular resistance, and increased blood pressure. However, RCR is related to blood pressure independent of type IIx myofiber % with a regression generated estimate of SBP and DBP (Fig. 2). So other factors other than type IIx myofiber % must be contributing to the link between mitochondrial coupling efficiency (RCR) and blood pressure.

Myofiber type is significantly related to SBP, but not DBP after adjusting for SVR. However, SVR is independently related to both SBP and DBP after adjusting for type IIa or type IIx myofiber %, suggesting that SVR affects blood pressure independent of fiber type (Fig. 2). Since many factors are known to affect SVR, this finding is not surprising.

Previous reports have shown that AA women had a higher percentage of type II myofibers (Tanner et al. 2002) and lower percentage of type I myofibers (Fisher et al. 2021). To our knowledge, this is the first study to find increased percentages of type IIx myofiber but decreased percentage of type IIa myofibers in AA women but not in type I myofibers. Both AA and EA women underwent exercise training and increased $\dot{V}{\text{O}}_{{{\text{2peak}}}}$, suggesting that exercise training did not play a role in race differences in percentage of type IIa and type IIx myofibers. Even though the participants trained at 80% of maximal heart rate for 40 min 3x/week for 12 weeks, it is possible the training volume/intensity may not have been sufficient to dismiss the racial differences in type IIx myofiber%. This extended training regime would also rule out any contributions of UCP3 since the activity is elevated upon acute exercise (Jones et al. 2003) and decrease after extended training (Fernström et al. 2004).

Body weight, BMI, fat free mass, CO, and SV have been shown to be related to blood pressure (Sidoti et al 2017), with fat free mass the strongest correlate. One factor that could be contributing to this relationship is uric acid levels that could be contributing to reduced renal function which in turn could result in increased blood pressure (Sidoti reference). To ensure that differences in weight, BMI, fat free mass, CO, and SV are not responsible for the racial blood pressure differences, we ran three separate ANCOVA with blood pressure as the dependent variable, race as the fixed factor, and weight, BMI, or fat free mass as the co-variate. AA women retained significantly higher blood pressures in all ANOVA analyses for SBP (all *p* < 0.001) and DBP (all *p* < 0.030). Analysis is shown in Tables 3, 4 for CO and SV.

These results support a model that shows that increased vascular resistance, increased mitochondrial coupling efficiency (RCR), AA heritage and type IIx myofiber% all contribute to increased blood pressure after a bout of high-intensity exercise (Fig. 4). Research suggests that aerobic exercise training can decrease systemic vascular resistance (Pagonas et al. 2017) and type IIx myofiber % (Owerkowicz et al. 2016), supporting the possibility that increased training (i.e., intensity, volume) may be beneficial for decreasing type IIx myofiber% and vascular resistance, thus decreasing blood pressure, at least in cases for which intensity and volume were too low to obtain maximal benefit.

We have previously found that RCR does not change with aerobic exercise training (Hunter et al. 2017). In addition, walking economy (i.e., net $\dot{V}{\text{O}}_{{{\text{2}}}}$) and $\dot{V}{\text{O}}_{{{\text{2max}}}}$ are inversely related (Borges et al. 2018). In addition, Broskey et al. (Broskey et al. 2014) showed that 16 weeks of endurance training increased oxidative capacity; however, there were no functional changes to oxidative coupling efficiency, suggesting that increases in oxidative capacity were due to mitochondrial biogenesis. Thus, at present, it remains unclear if exercise training can modify skeletal muscle mitochondrial efficiency.

## Data Availability

Data are not available.
